# Efficacy of Mobile phone use on adherence to Nevirapine prophylaxis and retention in care among the HIV-exposed infants in prevention of mother to child transmission of HIV: a randomized controlled trial

**DOI:** 10.1186/s12887-021-02660-w

**Published:** 2021-04-20

**Authors:** Lilian M. N. Kebaya, Dalton Wamalwa, Nyambura Kariuki, Bashir Admani, Philip Ayieko, Ruth Nduati

**Affiliations:** 1 Department of Paediatrics. Division of Neonatal-Perinatal Medicine, Western University, London, Ontario Canada; 2 Department of Paediatrics and Child Health, University of Nairobi, Nairobi, Kenya; 3 Department of Infectious Disease Epidemiology, London School of Hygiene and Tropical Medicine, London, UK

**Keywords:** Mobile phone use, Nevirapine prophylaxis, HIV-exposed infants, Prevention of mother to child transmission, PMTCT

## Abstract

**Background:**

HIV is a major contributor to infant mortality. A significant gap remains between the uptake of infant and maternal antiretroviral regimens and only a minority of HIV-exposed infants receives prophylaxis and safe infant feeding. Losses to follow-up of HIV-exposed infants are associated with shortcomings of facility-based PMTCT models with weak community support of linkages. Use of mobile phones offers an opportunity for improving care and promoting retention assessed by timely attendance of scheduled appointments for the mother-baby pairs and achievement of an HIV-free generation. The objective of this study was to compare self-reported adherence to infant Nevirapine (NVP) prophylaxis and retention in care assessed by timely attendance of scheduled appointments over 10 weeks in HIV exposed infants randomized to 2-weekly mobile phone calls (intervention) versus no phone calls (control).

**Methods:**

In this open label randomized controlled study, one hundred and fifty HIV infected women drawn from 3 health facilities in Western Kenya and their infants were randomly assigned to receive either phone-based reminders on PMTCT messages or standard health care messages (no calls) within 24 h of delivery. Women in the intervention arm continued to receive fortnightly phone calls. At 6- and 10-weeks following randomization we collected data on infant adherence to Nevirapine, mode of infant feeding, early HIV testing and retention in care in both study arms. All analyses were intention to treat.

**Results:**

At 6 weeks follow-up, 90.7% (*n* = 68) of participants receiving phone calls reported adherence to infant NVP prophylaxis, compared with 72% (*n* = 54) of participants in the control group (*p* = 0.005). Participants in the intervention arm were also significantly more likely to remain in care than participants in the control group [78.7% (*n* = 59) vs. 58.7% (*n* = 44), *p* = 0.009 at 6 weeks and 69.3% (*n* = 52) vs. 37.3% (*n* = 28), *p* < 0.001 at 10 weeks].

**Conclusions:**

These results suggest that phone calls are potentially an important tool to improve adherence to infant NVP prophylaxis and retention in care for HIV-exposed infants.

**Trial registration:**

PACTR202007654729602. Registered 6 June 2018 - Retrospectively registered, https://pactr.samrc.ac.za/TrialDisplay.aspx?TrialID=3449

## Background

HIV is a major contributor to infant mortality and in the absence of intervention, the risk of infant HIV acquisition from a HIV-positive mother is close to 40%. In 2018, an estimated 160,000 new HIV infections occurred among children under the age of 15, with 90% of these children getting the infection through vertical transmission [1, 2]. Cumulatively, globally nearly 3 million children are living with HIV and almost 250,000 HIV related deaths are reported yearly. Prevention of mother-to-child transmission is an important strategy in reducing infant mortality from HIV [1]. Kenya is one of the 22 priority countries for eliminating mother to child transmission of HIV [1, 3]. Despite overall progress, infant antiretroviral uptake significantly lags that seen in adult population and this may be attributable to poor retention and follow-up mechanisms within and outside health care systems [1].

There is growing evidence that mobile phones can improve antiretroviral therapy adherence among HIV-positive patients and improve health outcomes [4–7]. Information technology offers an opportunity to achieving an HIV-free generation through optimized communication and encouragement for retention of the mother-baby pairs. Expansion of mobile phone coverage in Africa, and in Kenya, where current coverage is 80%, presents an opportunity to scale up the HIV/AIDs response [8–10].

This study evaluated the effect of mobile phone use on adherence to Nevirapine prophylaxis, retention in care, early infant diagnosis (EID) and breastfeeding, using a randomized clinical trial design in Kisumu, Kenya. We recruited 150 mother infant pairs. We hypothesized that the phone call intervention would improve adherence and retention in PMTCT compared to the standard of care of giving an appointment at time of visit without follow-up reminders. Women in the intervention arm received phone calls fortnightly, while those in the control arm did not receive calls.

## Methods

### Study design

This open label randomized controlled trial used a parallel group design to individually randomize HIV positive women within 24 h of delivery to receive either phone-based reminders on PMTCT messages or standard health care messages (no calls), with an allocation ratio of 1:1. The study was conducted from 19th September 2013 to 31st January 2014. Participants were recruited at three health facilities in Kisumu, Western Kenya. These study sites were Jaramogi Oginga Odinga Teaching and Referral Hospital (JOOTRH), Kisumu East District Hospital (KEDH) and Lumumba Health Centre.

### Study eligibility

HIV infected women 18 years and older, who had a live birth, were eligible for enrollment if they owned a mobile phone on which they could receive calls, were willing to stay in the study area for at least 3 months after delivery and knew that they were HIV infected.

### Enrollment and randomization

On delivery, eligible women were invited to participate in the study after being provided detailed study information by trial staff. Women who agreed to participate signed a written consent and were interviewed using a standard tool to collect demographic, socioeconomic and biomedical data. Enrolled mothers were randomized into either intervention or control groups using computer generated block randomization sequence generated using STATA 9.0 software.

### Follow up procedures in the intervention arm

Every two weeks on a Monday morning, the researcher called each subject in the intervention arm until the infant was 10 weeks old. Each call was aimed at reminding them and reinforcing key PMTCT messages (Nevirapine prophylaxis, exclusive breastfeeding of the HIV-exposed infants, early infant diagnosis, scheduled immunizations) as well as ascertainment of their overall health. Calling was inexpensive (at 4 Kenya shillings per minute) lasting approximately 2–5 min per call. Study participants were also allowed to call to ask questions and report concerns on infant health. All mobile phone communications between clinicians and study participants were recorded in a study log.

### Follow up procedures in the control group

Participants randomized to the control arm received their usual standard of care (SOC) clinic support but were not called by the researcher. They were however free to call the researcher at any time of their own initiative.

### Follow-up activities in both arms

Study follow-up visits were designed to coincide with the scheduled well-baby follow-up clinics at six and 10 weeks of life. Data collection was conducted during the scheduled 6- and 10-week clinic appointments, using a standard tool. During these visits, information was collected on infant adherence to Nevirapine, retention in care and early infant diagnosis; and at 10 weeks, additional data was collected on breastfeeding practice.

### Ethics approval and consent to participate

The study was approved by the ethical review committees from the University of Nairobi, Kenyatta National Hospital and Jaramogi Oginga Odinga Teaching and Referral Hospital. All the women provided written informed consent prior to recruitment. Information about the purpose, procedures, risks and benefits of the study, as well as confidentiality and voluntariness of participation was provided to all potential participants as part of the informed consent process.

#### Data management

Data from the questionnaires were coded and entered into Microsoft Access 2007 database. Data entry and cleaning were conducted concurrently with data collection. SPSS version 17.0 was used to analyze data.

### Statistical analysis

The sample size determination showed that 75 participants per group allowed the study to detect a 50% relative difference in the primary outcome of retention in care at 6 weeks assuming a retention rate of 47% in the control arm, 95% level of confidence and 80% power.

Intervention and control arms of the study were compared using baseline characteristics. Similarities between the 2 groups was shown by comparing the baseline characteristics using Chi square/ Fishers’ exact tests and Student’s t / Mann Whitney U test for categorical and continuous variables respectively. Proportion of children that took their NVP as prescribed was used as the estimation of adherence. The prevailing guideline at the time of the study recommended infant NVP prophylaxis up to six weeks of age. The proportion of children seen at the well-baby clinic at six and ten weeks was the measure for retention in care. The proportion of HIV-exposed infants who had their HIV infection status determined by six and ten weeks was the estimate of EID. Infants in the two arms of the study were compared for adherence to NVP at six weeks, EID and retention in care at six and ten weeks, and prevalence exclusive breastfeeding at ten weeks. Logistic regression was used to estimate intervention effect using odds ratio (95% confidence intervals). All analyses were intention to treat and all statistical tests were based on a *p* value cut off of 0.05.

## Results

### Study population

**Fig. 1:**
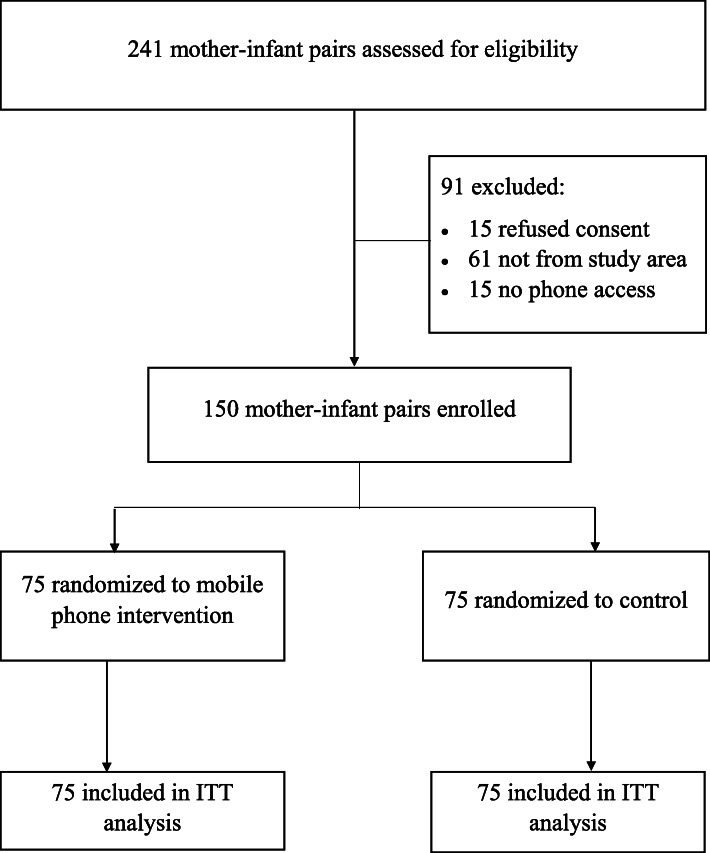
Flow diagram of mother-infant pair recruitment in study

A total of 241 mothers were screened, 91 were ineligible and 150 were enrolled in a randomized clinical trial, with 75 randomized to each arm (Fig. 1). Among the 91 excluded from the study, 15 refused consent, 61 were not from the catchments of the hospital and 15 did not have phone access.

The randomization process was successful at achieving comparability in the two arms of the study with regards to most maternal and infant socio-demographic and clinical parameters with the exception of employment and level of tertiary education as shown in Table 1. Compared to the intervention arm of the study, a higher proportion of women in the control arm were employed 42.7% versus 25.3%, and also had tertiary education 20% versus 8%. The odds of employment among mothers in the control group were 2-fold higher than for mothers in the intervention group (OR = 2.25, 95% CI 1.12–4.50, *p* = 0.025), while the odds of mothers in the control arm receiving tertiary education were 2-fold higher than for mothers in the intervention arm (OR = 0.35, 95% CI 0.1–1.02, *p* = 0.034). Participants in the intervention and control arms were comparable in key clinical parameters including proportion on HAART (68.9% for treatment compared to 58.7% in control), HIV disclosure to their partners (84.7% versus 83.3%) and proportion of partners who had tested for HIV (56% versus 58.7%) as shown in Table 1.

**Table 1:** Maternal and Newborn characteristics according to mobile phone intervention allocation

	Intervention (*n* = 75)		Control (*n* = 75)		*P* value
Characteristics	*n* (%)		*n* (%)		
**Maternal characteristics**					
Mean age in years (SD)*	25.9 (4.7)		26.9 (5.5)		0.2
Married	66	(88)	65	(86.7)	0.8
Employment status					
Employed	19	(25.3)	32	(42.7)	**0.02**
ANC attendance	74	(98.7)	75 (100)		0.3
Mother has other living children	55	(73.3)	53	(70.7)	0.7
**Education level**					
Tertiary	6	(8)	15	(20)	**0.03**
Secondary	24	(32)	21	(28)	0.6
Primary	44	(58.7)	39	(52)	0.412
None	1	(1.3)		0	NA
**Socioeconomic factors**					
Single roomed housing	35	(46.7)	37	(49.3)	0.7
Electricity within house	43	(57.3)	38	(50.7)	0.4
Cemented floor	21	(28)	21	(28)	1.0
**Newborn characteristics**					
Male	36	(50)	36	(50)	1.0
Mean birth weight (SD)	3104.1 (557)		3095.8 (529.8)		0.3
Place of birth					
KEDH	50	(66.7)	54	(72.9)	0.5
JOOTRH	15	(20)	15	(20)	1.00
Lumumba	9 (12)		5	(6.8)	0.3
Other	1	(1.3)		–	NA
Mode of delivery					
SVD	63	(84)	62	(82.6)	0.8
CS	12	(16)	12	(16)	1.00
**HIV-specific information**					
Currently on HAART	51	(68.9)	44	(58.7)	0.2
Currently on Option A	21	(28)	26	(34.7)	0.4
Mean CD4 count (SD)	396.6	(254.9)	443.1	(242.1)	0.3
HIV status disclosure to partner	61	(84.7)	60	(83.3)	0.8
Partner tested for HIV	42	(56)	44	(58.7)	0.7
Known HIV positive partner	31 (41.3)		31 (41.3)		1.0

*Option A:* mother took Zidovudine during the antenatal period, starting from as early as 14 weeks of pregnancy. A single dose of NVP and Lamivudine was added during labour, and Zidovudine and Lamivudine were continued for 7 days. If the mother breastfed, the baby received NVP syrup from birth until 1 week after all exposure to breast-milk had ended. If the mother was giving the baby replacement feeding, he or she would only get either NVP or zidovudine from birth until 6 weeks of age.

*Option B*: mother took a prophylaxis regimen consisting of 3 antiretroviral medicines during pregnancy, labour and after delivery until 1 week after all exposure to breast-milk had ended. Infants born to mothers on option B received either nevirapine or zidovudine from birth until 4–6 weeks of age, regardless of their feeding method. WHO recommended 4 possible triple antiretroviral prophylaxis regimens for option B, with the choice of regimen to be made at the country level.

#### Adherence to NVP prophylaxis at 6 weeks

**Fig. 2:**
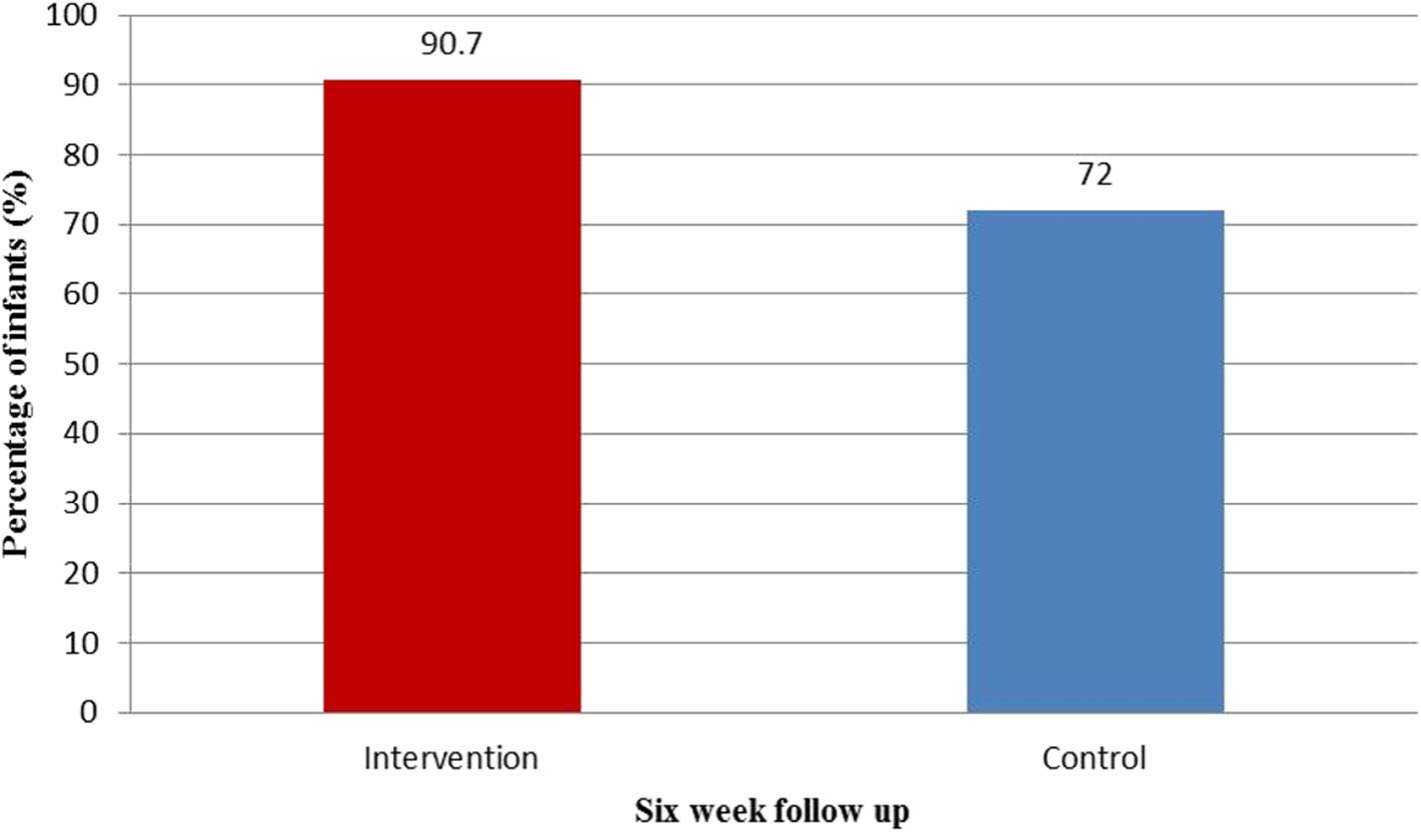
Adherence to infant Nevirapine prophylaxis

In the intervention arm of the study, 68 (90.7%) women reported that they had administered NVP to their babies compared to 54 (72%) of the women in the control arm of the study. Mothers in the intervention arm were three times more likely to report adherence to infant NVP prophylaxis (OR = 3.8, 95% CI 1.5–9.5, *p* = 0.005) [shown in Fig. 2]. As per prevailing guidelines, recommended infant NVP prophylaxis was up to 6 weeks of age. We did not report the 10 weeks results as only 47 mothers who were on Option A were analyzed. The majority of the mothers were on HAART and their infants stopped taking NVP by six weeks.

#### Retention in care at 6 and 10 weeks

**Fig. 3:**
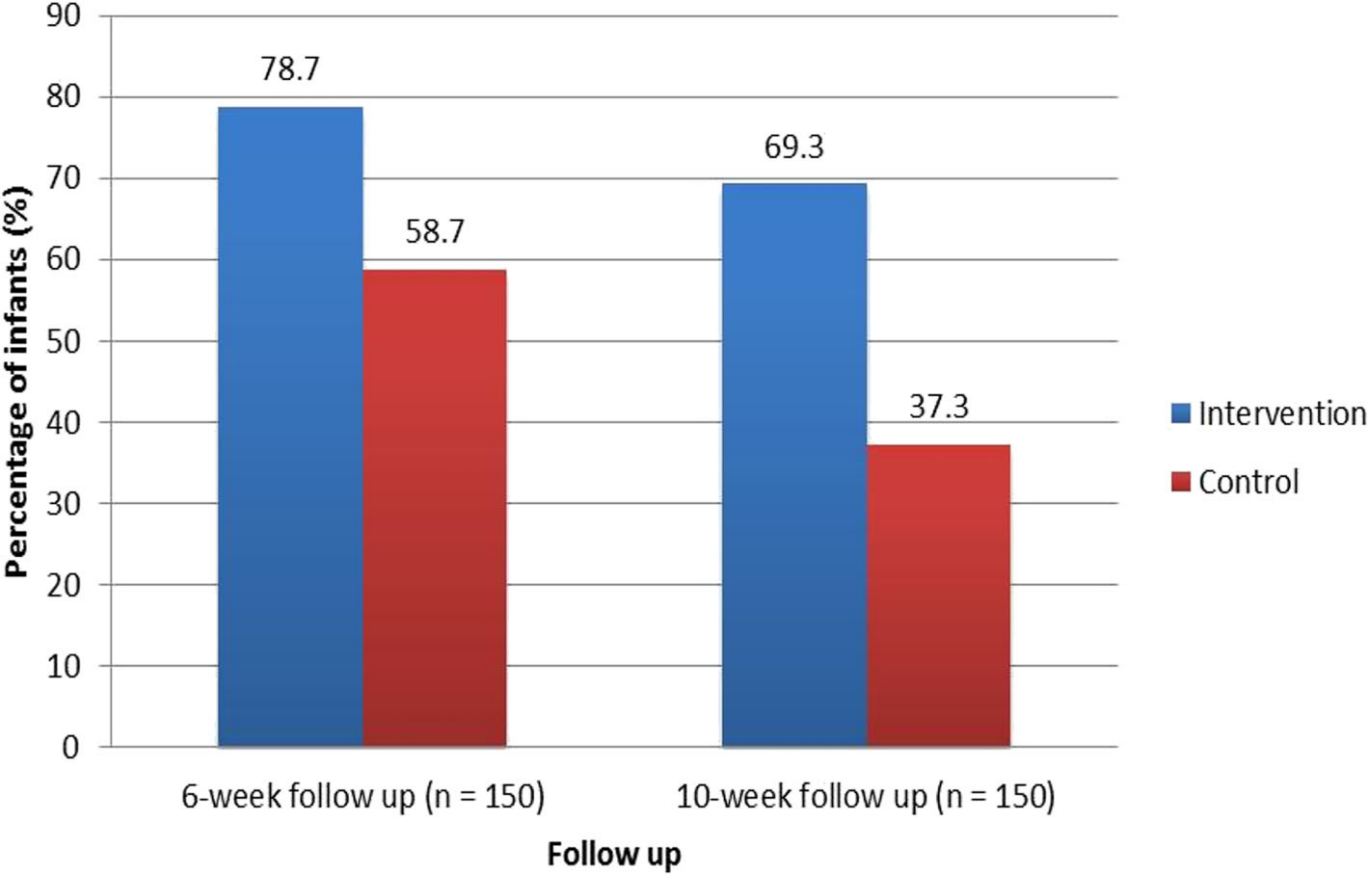
Retention in care

At six weeks 59 mother-infant pairs (78.7%) in the intervention arm and 44 (58.7%) mother-infant pairs in the control arm attended clinic as scheduled. Mother-infant pairs in the intervention arm of the study had a greater than 2-fold increased likelihood of retention in care at six weeks post-delivery (OR = 2.6, 95% CI 1.3–5.3, *p* = 0.009) [Fig. 3]. At ten weeks 52 mother-infant pairs (69.3%) in the intervention arm and 28 mother-infant pairs (37.3%) in the control arm were still in care. Up to 30% of the mother-infant pairs in the intervention arm and 62% in the control arm were no longer in care in the facility where the women delivered. (OR = 3.8, 95% CI 1.9–7.5, *p* < 0.001).

#### Early infant diagnosis (EID)

Timing of early infant diagnosis was defined as a test done, preferably by the 6 weeks’ clinic visit, and at subsequent visits for those that missed out. At six weeks, 53 infants (70.7%) in the intervention arm had a PCR taken for EID compared to 44 (58.7%) in the control arm. At the tenth week, only one additional child in the intervention arm and two in the control arm had shown up for EID. Overall 54 (72%) of 75 infants in the intervention arm, compared to 46 (61.3%) in the control arm had received PCR testing. The odds of EID testing at six and ten weeks were not significantly different between the intervention and control groups [week 6: OR = 1.7 (0.9–3.3) and week 10: OR = 1.6 (0.8–3.2)]. At the facility, there were missed opportunities for EID. At the sixth week visit, 5 (8.4%) of the 59 infants in the intervention arm of the study showed up for the visit-missed EID. All 44 infants in the control arm were tested.

#### Infant feeding

Among the women still in care at ten weeks the rate of reported exclusive breastfeeding was 97% in the intervention group compared to 90.7% in the control group, OR = 3.8 (0.8–18.7), *p* = 0.1. Table 2 presents the analysis of impact of mobile phone intervention on secondary outcomes (EID and infant feeding).

**Table 2:** Secondary outcomes

	Intervention	Control	OR (95% CI)		***P*** value
**Early infant diagnosis**					
6 weeks (*n* = 150)	53 / 75 (70.7)	44 / 75 (58.7)	1.7	(0.9–3.3)	0.126
10 weeks (*n* = 150)	54 / 75 (72.0)	46 / 75 (61.3)	1.6	(0.8–3.2)	0.167
**Exclusive breastfeeding**					
10 weeks (*n* = 150)	73 / 75 (97.3)	68 / 75 (90.7)	3.8	(0.8–18.7)	0.106

## Discussion

There are three key findings from this study. The first is that mobile phone reminders improved adherence to infant NVP prophylaxis. At six weeks, 90.7% of the infants in the intervention arm were reported to have received the NVP prophylaxis, compared to 72% in the control arm of the study, a 19% difference. Assuming HIV transmission rate of 20% at six weeks for babies who did not access NVP prophylaxis, assuming a population of 100 HIV-exposed infants, the phone intervention would have averted four infant infections. The rate of adherence to infant NVP prophylaxis was higher than national data which demonstrated that only 63% of exposed infants receive ARV prophylaxis at six weeks [11]. The results of this study have important implications as Kenya strives to meet its stated goals of elimination of MTCT [12].

The second important finding was that the fortnightly reminders improved retention at both times of observation. At six weeks 78.7% of the mother-infant pairs in the intervention arm were still in care compared to 58.7% in the control arm, a 20% difference in retention in care. Put in another way, four out of five HIV-exposed infants in the intervention arm were still in care compared to just less than three of five in the control arm of the study. Mobile phone calls have been shown to increase ARV adherence and retention in care among adult populations, however, there is limited data regarding this strategy in the context of prevention of mother to child transmission of HIV or HIV-exposed infant care. Our study is an important contribution towards improved quality of care for HIV-exposed infants. The findings of this study are comparable to a Kenyan study in which short message service (SMS) reminders improved adherence to ART among patients attending a rural clinic (90% after 48 weeks of follow-up among those in the intervention arm compared to 40% among the controls) [6].

A third observation was that there was a strong trend towards higher rates of early infant diagnosis in the intervention arm (70.7% versus 58.7% at six weeks). The rate of EID in this study is higher than the national rate of 35% [11] and this could be attributed to the controlled study setting. On exclusive breastfeeding, although the results were not statistically significant at 10 weeks, we showed a difference between the two groups (97.3% compared to 90.7% = 0.1). This was a relatively small study and with a larger study we might be able to show a difference. This could be attributed to the level of counseling in the PMTCT program.

Our study involved direct phone calls in which the mother had the opportunity to ask questions including those related to their child’s health. A previous study at a Kenyan tertiary facility, demonstrated that weekly phone calls during the six postpartum weeks to primiparous mothers improved infant survival at six and 10 weeks (Kihara). Retention in care for HIV exposed infants remains an important challenge with estimates in Kenya still largely unknown. Although our study did not follow infants through the first year of life, the findings suggest that even as early as 10 weeks, retention was impacted by regular phone reminders. These results suggest that there is potential to use this tool for other health related aspects.

Randomization was successful, and the two groups were comparable on most characteristics, however women in the control arm were more likely than those in the intervention arm to be employed (2 fold higher) and have received tertiary education (2 fold higher). Both of these factors are well recognized drivers of good health seeking behavior [13, 14]. Consequently the relative imbalance on the two characteristics could have attenuated our findings as a mother’s education can exert a positive influence on her children’s health and survival and a more educated mother is more likely to adhere to instructions than an uneducated one.

There were a number of limitations to our study. There might have been an overestimate to report ART adherence since the study relied on self-reports. Facility level adherence data such as pharmacy refill to verify adherence to NVP prophylaxis in infants and infant HIV status were not ascertained. Nevertheless, there was strong corroboration between the rate of adherence and retention in care suggesting that in this study self-reports were reasonably accurate. Another limitation was the customary use of mobile phones by more than one individual in some homes which poses a confidentiality risk and which may impact on continuity of care. It is illustrative that although retention in the intervention arm of the study was significantly higher, 29% of the women were lost to follow-up by the tenth week of the postnatal visit. This may have resulted in differential loss to follow-up given that mobile phone ownership is related to socioeconomic status. Further research on mobile phone use will need to continue to address issues of confidentiality.

Overall, these results suggest that phone calls can be an important tool to improve adherence to infant NVP prophylaxis and retention in care for HIV-exposed infants. Given the high rate of mobile phone penetration (above 80%) there is potential to scale up this intervention to other parts of the country and region. The ART regimen for PMTCT changed from Nevirapine to a two-dose regimen in 2016. Despite the regimen changes after completion of the findings reported in the current study, the problem of non-adherence to ART regimens in PMTCT remains and evidence on effective approaches for improving ART adherence through patient follow up is still needed. Mobile phone coverage has increased consistently including in the period following study completion. In this regard, the evidence presented in this study remains relevant to PMTCT care given that ART has been retained as the mainstay of PMTCT care and mobile phone coverage is increasing. A key concern however is that the poorest sectors of the population either may not afford a mobile phone, or will share one handset leading to unique challenges in rolling out m-health interventions such as the one evaluated here. In addition, cost effectiveness studies will be critical in understanding the true benefits and best implementation strategies of mobile phone use for follow-up in PMTCT programs.

## Conclusions

These results suggest that phone calls are potentially an important tool to improve adherence to infant NVP prophylaxis and retention in care for HIV-exposed infants.

## Data Availability

The datasets during and/or analyzed during the current study available from the corresponding author on reasonable request.

## Abbreviations

AIDS: Acquired Immune Deficiency Syndrome
ART / ARV: Antiretroviral Therapy / Antiretrovirals
CD4: Cluster of Differentiation 4
EID: Early Infant Diagnosis
HAART: Highly active antiretroviral therapy
HIV: Human Immunodeficiency Virus
JOOTRH: Jaramogi Oginga Odinga Teaching and Referral Hospital
KEDH: Kisumu East District Hospital
NVP: Nevirapine
MCH: Maternal and Child Health
PMTCT: Prevention of Mother-to-Child Transmission of HIV
SD: Standard Deviation
UN: United Nations
WHO: World Health Organization
